# Seroprevalence of Chikungunya Virus in a Rural Community in Brazil

**DOI:** 10.1371/journal.pntd.0005319

**Published:** 2017-01-20

**Authors:** Rivaldo V. Cunha, Karen S. Trinta, Camila A. Montalbano, Michel V. F. Sucupira, Maricelia M. de Lima, Erenilde Marques, Izilyanne H. Romanholi, Julio Croda

**Affiliations:** 1 Faculty of Medicine, Federal University of Mato Grosso do Sul, Campo Grande, Mato Grosso do Sul, Brazil; 2 Oswaldo Cruz Foundation, Campo Grande, Mato Grosso do Sul, Brazil; 3 Bio-Manguinhos, Oswaldo Cruz Foundation, Rio de Janeiro, Brazil; 4 Faculty of Medicine, Federal University of Mato Grosso do Sul, Campo Grande, Mato Grosso do Sul, Brazil; 5 Municipal Health Department of Feira de Santana, Feira de Santana, Bahia, Brazil; 6 State University of Feira de Santana, Feira de Santana, Bahia, Brazil; 7 Falcuty of Health Sciences, Federal University of Grande Dourados, Dourados, Mato Grosso do Sul, Brazil; Centers for Disease Control and Prevention, Puerto Rico, UNITED STATES

## Abstract

**Background:**

The emergence of the Chikungunya virus (CHIKV) is currently expanding. In 2015, 38,332 cases of Chikungunya were reported to the Brazilian epidemiological surveillance system. Eighteen months after notification of the first case in the city of Feira de Santana, we conducted the first serosurvey to define the magnitude of transmission in a rural community in Brazil.

**Methodology/Main findings:**

The serosurvey was conducted in a random sample of 450 residences in the Chapada district, located 100 kilometers from Feira de Santana. We administered questionnaires and tested 120 sera from Chapada district residents for CHIKV IgM- and IgG-specific antibodies. An individual with CHIKV infection was defined as any person with CHIKV IgM or IgG antibodies detected in the serum. One Hundred cases of Chikungunya were reported after prolonged rainfall, which reinforced the relationship between the rainfall index and CHIKV transmission. Eighteen months after the start of the outbreak, we identified a seroprevalence of 20% (95% CI, 15.4–35%). CHIKV IgG- and IgM-specific antibodies were detected in 22/120 (18.3%) and 6/120 (5.0%) individuals, respectively. Among seropositive patients, 13/24 (54.2%) reported fever and joint pain over the previous two years (p<0.01). The rate of symptomatic CHIKV infection was 40.7%.

**Conclusions/Significance:**

We identified a moderate seroprevalence of Chikungunya in the Chapada district, and in half of the confirmed CHIKV infections, patients reported arthralgia and fever over the previous two years.

## Author Summary

The Chikungunya virus (CHIKV) epidemic is currently expanding. In 2015, 38,332 cases of Chikungunya were reported to the Brazilian epidemiological surveillance system. To date, no serosurvey has described the impact of the disease on rural communities in Brazil. We conducted a survey in which households in the Chapada district, located in Riachão do Jacuípe in Bahia, were randomly selected for serum collection to perform a CHIKV antibody test. More than 100 cases of Chikungunya were reported in the Chapada district following a three-month period of rain. Eighteen months after the start of the outbreak, we identified that 20% of the population had acquired CHIKV, and 13/24 (54.2%) reported fever and joint pain over the previous two years. The rate of symptomatic CHIKV infection was 40.7%. We identified a moderate seroprevalence of Chikungunya in the Chapada district, and half of the patients reported arthralgia and fever over the previous two years.

## Introduction

The Chikungunya virus (CHIKV) belongs to the genus *Alphavirus* of the family *Togaviridae* and has a single-stranded RNA genome and positive polarity [1]. The virus was first isolated in blood samples obtained during an epidemic of a "dengue-like" disease that occurred between 1952–1953 in Tanzania [2, 3]. To date, four CHIKV genotypes have been identified, two of which were initially isolated in Africa: the East-Central-South-African genotype (ECSA), the West African genotype, the Asian genotype and the more recently identified Indian Ocean Lineage [4, 5].

Clinical disease manifestations emerge after an incubation period that lasts an average of 2 to 4 days. The first symptom is usually a high fever, followed hours later by myalgia and generalized arthralgia and arthritis, which are often incapacitating and accompanied by headache and back pain. Polyarthralgia/polyarthritis is usually bilateral and symmetrical and occurs more often in the hands, wrists, interphalangeal joints, feet and ankles but may also affect large joints, such as the shoulders and knees. Periarticular swelling is frequently observed [6, 7]. A maculopapular rash and facial swelling are present in approximately 40 to 50% of patients [8]. In children, vesicobullous eruptions with intense subsequent flaking may occur, along with petechiae and gingivorrhagia. Ocular manifestations can also occur and generally achieve satisfactory resolution in six to eight weeks [6, 9]. Chikungunya is usually self-limiting, with clinical manifestations regressing within a few weeks. However, in a percentage of those infected, which can vary from 30 to 40%, polyarthralgias may persist for months or even years [7].

Although Chikungunya is usually benign, there have been increasingly frequent reports associating Chikungunya with the decompensation of pre-existing diseases, especially heart, kidney and liver diseases, diabetes, hypertension and systemic lupus erythematosus, among others [10]. During the epidemics in the Caribbean and Americas between 2013 and 2014, 65 patients were admitted to intensive care units (ICUs) with Chikungunya, of whom 54 had pre-existing illnesses, and 27 were admitted due to the exacerbation of comorbidities [11].

In October 2013, autochthonous CHIKV circulation was found on the island of Saint Martin and spread to different countries and territories of the Caribbean and the Americas [12]. In Brazil, the first autochthonous cases of CHIKV were identified in the cities of Oiapoque, Amapá State and Feira de Santana, Bahia State, in September 2014 [4]. The Asian genotype, which had already been isolated in Caribbean and Central American countries and territories, was identified in the first location. In the city of Feira de Santana, the detected genotype was ECSA [4, 13]. Between late 2013 and July 2016, approximately two million cases were reported in more than forty countries and territories in the Americas and the Caribbean, with Brazil also having registered tens of thousands of cases [14].

A Chikungunya epidemic began in Riachão do Jacuípe, Bahia in the second half of 2014 [15]. Chapada is a district in Riachão do Jacuípe that reported a high number of cases in 2014 and 2015. The Chapada district is a small, restricted geographic region with 100% basic health care coverage, which would facilitate the performance of a seroepidemiological survey. Therefore, to determine the prevalence of anti-CHIKV antibodies after the first wave of the epidemic, a serosurvey was conducted in the District of Chapada, a rural community of Riachão do Jacuípe.

## Methods

### Study location

Riachão do Jacuípe has an area of 1,190.215 km^2^ and is located 100 km from the city of Feira de Santana, where the first case was identified in Northeastern Brazil. It has an estimated population of 33,000 inhabitants and a Human Development Index (HDI) of 0.628. The Chapada district is 17 km from the administrative headquarters of Riachão Jacuípe and has a population of 2,303 inhabitants [16]. The population is mainly rural, and farm work is the main economic activity of its inhabitants.

### Study design and sample selection

This serosurvey was conducted in April 2016. The Chapada district has a total of 505 households, with 2,303 individuals. Cluster sampling was performed, using the household as a sample unit. To calculate the sample size, we estimated a prevalence of 20% with a variation of ± 10, 80% power, type I error of 5%, and a correction for survey design by an aggregate of 2. Therefore, it was necessary to select 120 individuals, increasing this number by 20% for losses. We conducted a random selection of 45 households and selected 160 individuals to compose the sample.

### Laboratory diagnosis and definition of CHIKV infection

Blood samples were collected by venipuncture, and serum was separated by centrifugation (3.000 rpm/5 minutes). The serum was stored at—20°C until use. The search for anti-CHIKV IgM- and IgG-specific antibodies was performed using a commercial ELISA (enzyme-linked immunosorbent assay) according to the manufacturer's instructions (Euroimmun, Lübeck, Schleswig-Holstein, Germany). An individual with CHIKV infection was defined as any person with IgM or IgG CHIKV antibodies detected in the serum.

### Definition of symptomatic individuals and rate of symptomatic CHIKV infection

An individual was considered to have had symptomatic infection if he/she had experienced fever and arthralgia in the previous two years and if a test for IgM and/or IgG detection had yielded positive results. To calculate the symptomatic rate of Chikungunya, we subtracted the rate of individuals who had fever, arthralgia and negative CHIKV serology from the rate of individuals who had fever, arthralgia and positive CHIKV serology.

### Secondary sources of data

Chikungunya is a mandatory reportable disease, and the reporting system relies on the notification of all suspected cases at public and private health facilities based on the attending clinician’s initial clinical diagnosis (not laboratory confirmed). A suspected case is defined as illness in a person from an area of Chikungunya transmission or Aedes aegypti mosquito infestation who has symptoms of Chikungunya (sudden onset fever greater than 38.5°C and arthralgia or acute arthritis, unexplained by other conditions). The Sistema Nacional de Informação do Ministério da Saúde (SINAN) is the Brazilian Ministry of Health's National Information System for entering and processing the data for reported Chikungunya cases throughout Brazil, and the notification rate is calculated by dividing the number of Chikungunya cases reported to SINAN by the total population.

### Data analysis and ethical considerations

The interviews were conducted with the aid of a notebook. Data were entered directly into Research Electronic Data Capture (REDCap), hosted at the Federal University of Grande Dourados [17], and were analyzed using the statistical program SAS 9.2 (SAS Institute, Cary, NC, USA). The incidence and prevalence of Chikungunya are expressed as percentages with 95% confidence intervals. Dichotomized and categorical data were analyzed with the chi-squared test. The following data were collected on the questionnaire: age, sex, educational attainment, previous fever or joint pain in the last two years and medical history of dengue, Chikungunya and Zika. The participant’s race/ethnicity (i.e., white, black, indigenous, Asian or mixed) was self-reported. All eligible participants provided written informed consent prior to study participation. The study was approved by the Research Ethics Committee at the State University of Feira de Santana (CAAE study number: 49946515.6.0000.0053. Institutional review board approval number: 1.450.762).

## Results

**Fig 1:**
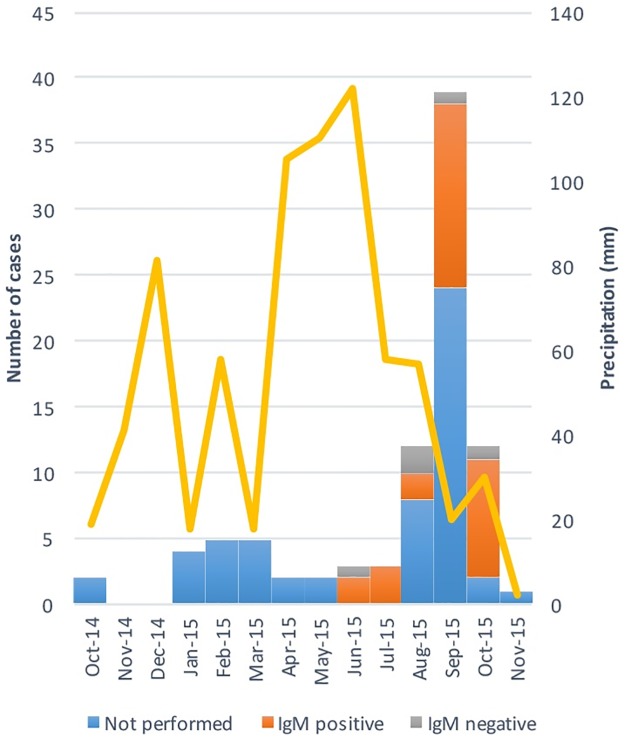
Numbers of CHIKV cases, anti-CHIKV IgM status and monthly precipitation in the Chapada district between October 2014 and November 2015.

The first suspected case of Chikungunya in the Chapada district was reported in October 2014. By October 2015, over 100 cases had been reported, with 35% serologically confirmed and 30 anti-CHIKV IgM-positive cases and 5 anti-CHIKV IgG-positive cases (Fig 1). The notification rate over the one-year period was 4.3% (95% CI, 3.5%-5.3%), and all of the reported cases involved joint pain and fever. The notification rate in in the Chapada district was similar from the notification rate over the same period in Riachão do Jacuípe (6.7%; 95% CI, 6.4%-7.0%).

**Fig 2:**
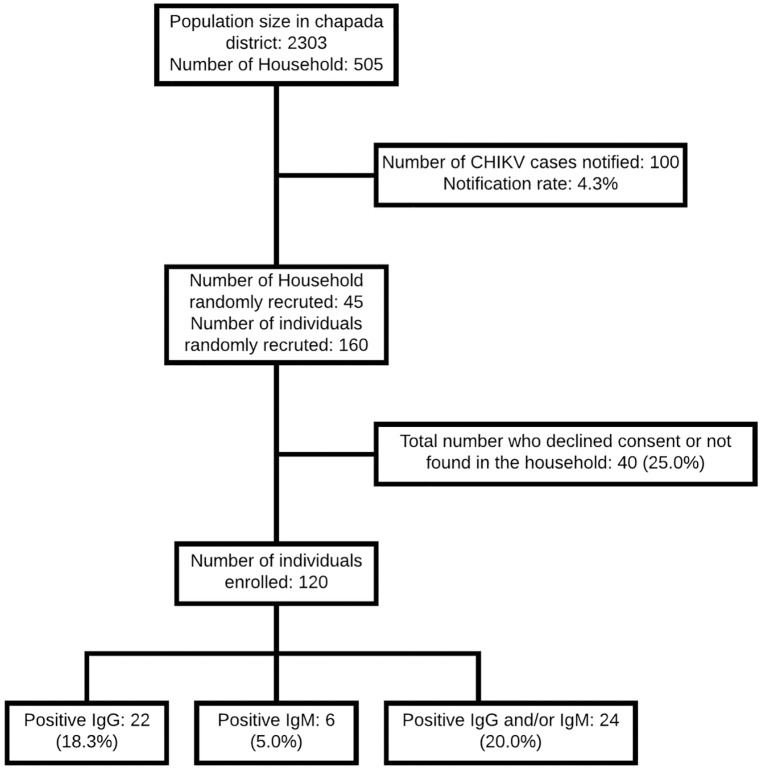
Flow chart of the study enrollment and CHIKV IgM and IgG antibody positivity rates.

In the serosurvey, of the 160 individuals selected, 120 were present in their homes and agreed to participate in the study (Fig 2). Randomly recruited individuals did not significantly differ from the general population of the Chapada district with respect to age and gender (S1 Table).

Differences in the age and sex of the individuals who agreed and declined to participate in the study were not observed. Most individuals were female, 62/120 (51.7%), and young, with a mean age of 36.6±20.9, were black, 45/117 (38.5%), or of mixed race, 69/117 (59.0%), and were not working, 30/120 (25%), at the time of the survey. Of the individuals evaluated, 15/120 (12.5%) reported a previous clinical diagnosis of Chikungunya, and 44/120 (36.7%) and 30/120 (25.0%) reported at least one episode of fever or joint pain, respectively, in the previous 2 years (Table 1).

**Table 1:** Description of 120 individuals enrolled in the serosurvey in the Chapada district.

Characteristics	N (%)
**Sociodemographics**	
**Sex, Female**	62 (51.7%)
**Age groups (years)**^1^	
0–14	22 (18.5%)
15–29	30 (25.2%)
30–44	28 (23.5%)
45–59	20 (16.8%)
≥60	19 (16.0%)
**Race**^2^	
White	3 (2.6%)
Mixed	69 (59.0%)
Black	45 (38.5%)
**Less than 4 years of schooling**	53 (44.2%)
**Occupation**	
Unemployed	30 (25.0%)
Student	30 (25.0%)
Retired	14 (11.7%)
Rural work	16 (13.3%)
Others	30 (25.0%)
**Clinical symptoms**	
**Fever in the last 2 years**	44 (36.7%)
**Joint pain in the last 2 years**	30 (25.0%)
**Prior clinical diagnosis in the last 2 years**	
Dengue	7 (5.8%)
Zika	6 (5.0%)
Chikungunya	15 (12.5%)

^1^ Information available for 119 individuals

^2^ Information available for 117 individuals

Among the 120 individuals evaluated, 22/120 (18.3%) were anti-CHIKV IgG-positive, and 06/120 (5.0%) were anti-CHIKV IgM-positive. Three samples were borderline on the test used. Among the six patients who had CHIKV-specific IgM, four (66.7%) also showed CHIKV specific-IgG antibodies, for an overall prevalence of 20% (95% CI, 15.4–35.7%) (Table 2).

**Table 2:** Seroprevalence of IgM and IgG antibodies against CHIKV in Chapada, April 2016 (N = 120).

	IgG	IgM	IgG and/or IgM
**Positive**	22 (18.3%)	6 (5.0%)	24 (20.0%)
**Negative**	98 (81.7%)	111 (92.5%)	94 (78.3%)
**Borderline**	0 (0%)	03 (2.5%)	02 (1.7%)

Among the patients with positive serology, 16/24 (66.7%) suffered fever episodes, and 13/24 (54.2%) reported joint pain and fever over the previous two years (p<0.01). Among 96 patients with negative serology, 13 (13.5%) had fever and joint pain. According to the criteria defined for this study, the rate of symptomatic CHIKV infection was 40.7%. Furthermore, 54.2% reported having had a clinical diagnosis of Chikungunya (p<0.01) (Table 3).

**Table 3:** Comparison of individuals with CHIKV IgG- and/or IgM-positive and negative antibodies in the Chapada district (N = 120).

	Positive (N = 24)	Negative (N = 96)	P value
**Sex, Female**	10 (41.7%)	52 (54.2%)	0.27
**Age groups (years)**^1^			0.94
0–15	3 (13.0%)	19 (19.8%)	
>15–30	7 (30.4%)	23 (24.0%)	
>30–45	6 (26.1%)	22 (22.9%)	
>45–60	4 (17.4%)	16 (16.7%)	
>60	3 (13.0%)	16 (16.7%)	
**Race**^1^			0.74
White	0 (0.0%)	3 (3.1%)	
Mixed	15 (65.2%)	54 (56.3%)	
Black	8 (34.8%)	37 (38.5%)	
**Less than 4 years of schooling**	8 (33.3%)	45 (46.9%)	0.23
**Fever in the last 2 years**	16 (66.7%)	28 (29.2%)	<0.01
**Joint pain in the last 2 years**	13 (54.2%)	17 (17.7%)	<0.01
**Fever and joint pain in the last 2 years**	13 (54.2%)	13 (13.5%)	0.01
**Prior clinical diagnosis in the last 2 years**			
Dengue	0 (0%)	7 (7.3%)	0.40*
Zika	2 (8.3%)	4 (4.2%)	0.69*
Chikungunya	13 (54.2%)	2 (2.1%)	<0.01*

^1^Information available for 23 individuals

^2^Fisher's exact test

## Discussion

This report describes the first serosurvey in a rural community in Northeastern Brazil. The seroprevalence found in the Chapada district was 20%, in other serosurveys, the prevalence of anti-CHIKV antibodies has ranged between 10.2% and 75% [18, 19]. The difference between the prevalence found in this study and those reported by other serosurveys may be associated with local environmental conditions and/or vector competence in transmitting the ECSA strain of CHIKV, which was introduced in Brazil in 2013 [13]. In addition, the interval between the time the serosurvey was conducted and the peak of outbreak could have affected the prevalence rates reported in different cross-sectional studies [18, 20, 21].

The Chapada district has a population of 2,303, and during the year in which the outbreak occurred, 100 Chikungunya cases were reported. The attack rate found in the Chapada district may eventually be applied to Riachão do Jacuípe and other cities that present climatic conditions and a similar Aedes infestation index in Brazil. Based on the number of notifications and serosurvey results, we can estimate that for every reported case, 1.9 cases of symptomatic CHIKV infection were not reported during the outbreak, demonstrating the difficulty that the surveillance system faces in identifying suspected Chikungunya cases. Nevertheless, the underreporting rate is still much lower than that recorded for dengue, wherein for each case reported, 12 are not reported in the Brazilian Ministry of Health's National Information System [22].

The Chapada district is located in a semi-arid region of Bahia, 100 km from Feira de Santana, where the first case of Chikungunya was identified in Brazil [4]. Interestingly, the peak in Chikungunya outbreak notifications occurred in September 2015 and was preceded by a very rainy period between April and June 2015. This condition may have been crucial to the reproduction and multiplication of the *Aedes* vector and may consequently have affected the occurrence of the outbreak in 2015. It must be taken in account that environmental factors are important in the occurrence of Chikungunya outbreaks. Studies conducted on the island of Saint Martin [18] and in Feira de Santana [23] and mathematical modeling studies conducted in American and Asian countries clearly demonstrate the relationship between temperature, rainfall and the increase in the number of Chikungunya notifications [24].

The majority of randomly selected individuals were unemployed, children and retirees (61.7%), and half of the population had less than four years of schooling 44.2% (53/120). Of the 24 positive cases, 54.2% (13/24) reported significant arthralgia in the previous two years, and the rate of symptomatic Chikungunya infection was 40.7%. Chikungunya epidemics have become a serious public health problem, especially in the poorest communities, where health care systems are more precarious. The broad spectrum of post-Chikungunya musculoskeletal and rheumatic disorders, which can evolve chronically and are often disabling, [25] compromise the local work force and affect the family incomes of these most vulnerable populations.

Arthralgia is one of the Brazilian Ministry of Health’s clinical criteria for case definition. Despite the fact that all 100 reported patients during the outbreak experienced this symptom, only 54.2% of patients with CHIKV-specific antibodies in the serosurvey reported the presence of this symptom in the previous 2 years, and 54.2% reported having had a clinical diagnosis of Chikungunya. Similar frequencies of patients without arthralgia have also been described in the ECSA lineage outbreaks that occurred in Thailand, Kenya and Saint Martin [18, 21, 26]. Asymptomatic and oligosymptomatic patients are fundamental to the maintenance of a local epidemic. There is always the possibility of recall bias in serosurveys. However, Chikungunya-induced arthralgia is usually marked; thus, we believe that information obtained retrospectively would not have suffered from any study design effect.

This study has important limitations inherent to its design. A major example of these limitations is that 40 individuals (25%) were not at home, which can lead to selection bias, as demonstrated by the higher proportions of women, children and elderly. Nonetheless, we observed no differences in relation to age and sex between the individuals who agreed and declined to participate in the study and compared to the general population.

In summary, the incidence of CHIKV infection during an outbreak in the Chapada district was 20% (95% CI 15.4–35.7%). The outbreak was preceded by a 3-month rainy period, which is unusual for this district and which may have affected the reproduction and multiplication of the *Aedes* vector. In addition to the substantial suffering associated with persistent arthralgia, the outbreak in this community of rural workers likely had a significant economic impact on the region.

## Supporting Information

S1 Table Comparison among the individuals enrolled in the serosurvey and the general population in the Chapada district. (DOCX)

S1 Dataset CHIKVchapada. (CSV)

S1 Checklist (DOC)
