# Supplementary material for: Seroprevalence of Chikungunya Virus in a Rural Community in Brazil
Source: PLoS Negl Trop Dis. 2017 Jan 20;11(1):e0005319. doi: 10.1371/journal.pntd.0005319 (PMC5287455; doi:10.1371/journal.pntd.0005319)
Supplement: S1 Table — (DOCX) [file pntd.0005319.s001.docx]

|  | **Sample (N=120)** | **General population (N=2,303)** | **P Value^1^** |
| --- | --- | --- | --- |
| **Characteristics** | **N (%)** | **N (%)** |  |
| **Sociodemographics** |  |  |  |
| **Sex, Female** | 62 (51.7%) | 1152 (50%) | 0.72 |
| **Age groups (years)^1^** |  |  | 0.47 |
| 0-14 | 22 (18.5%) | 570 (24.8%) |  |
| 15-29 | 30 (25.2%) | 619 (26.9%) |  |
| 30-44 | 28 (23.5%) | 457 (19.8%) |  |
| 45-59 | 20 (16.8%) | 318 (13.8%) |  |
| ≥60 | 19 (16.0%) | 339 (14.7%) |  |
| ^1^ chi-square test |  |  |  |
